# Structural response of microtubule and actin cytoskeletons to direct intracellular load

**DOI:** 10.1083/jcb.202403136

**Published:** 2024-11-15

**Authors:** Ryota Orii, Hirokazu Tanimoto

**Affiliations:** 1 Department of Science, Yokohama City University, Yokohama, Japan

## Abstract

Microtubule and actin are the two major cytoskeletal polymers that form organized functional structures in the interior of eukaryotic cells. Although the structural mechanics of the cytoskeleton has been extensively studied by direct manipulations in in vitro reconstitution systems, such unambiguous characterizations inside the living cell are sparse. Here, we report a comprehensive analysis of how the microtubule and actin cytoskeletons structurally respond to direct intracellular load. Ferrofluid-based intracellular magnetic tweezers reveal rheological properties of the microtubule complex primarily determined by filamentous actin. The strain fields of the microtubule complex and actin meshwork follow the same scaling, suggesting that the two cytoskeletal systems behave as an integrated elastic body. The structural responses of single microtubules to contact and remote forces further evidence that the individual microtubules are enclosed by the elastic medium of actin. These results, directly characterizing the microtubule and actin cytoskeletons as an interacting continuum throughout the cytoplasm, serve as a cornerstone for the physical understanding of intracellular organization.

## Teaser

Orii and Tanimoto study the structural mechanics of microtubule and actin cytoskeletons using high-force intracellular magnetic tweezers. The measurements directly reveal the integrated nature of the two cytoskeletons throughout the cytoplasm, which defines their physical behavior as a simple cell-scale continuum.

## Introduction

Microtubule and actin represent protein polymers termed the cytoskeleton that forms organized active structures inside the living cell. The cytoskeleton has diverse physical functions. For instance, the very rigid microtubules compose an astral structure that stabilizes organelles (Bornens, 2008), and the semi-flexible actin filaments compose a dense network that generates cellular forces (Stricker et al., 2010). Measuring the physical properties of the cytoskeleton at work is a key to understanding their physiology. However, in situ physical characterization of the cytoskeleton, as well as of other intracellular structures, remains a major challenge in cellular biophysics (Anjur-Dietrich et al., 2024; Garzon-Coral et al., 2016; Goda et al., 2024, *Preprint*; Long et al., 2020; Tanimoto et al., 2018).

The relationship between the force and structure is a fundamental property which defines the physical behavior of any matter. The force–structure relationship of the microtubule and actin cytoskeletons has been studied predominantly by applying external forces to the cell surface, by means of microneedles (Heidemann et al., 1999; Lombardi et al., 2011; Maniotis et al., 1997; Wang et al., 2001), substrate stretch (Jungbauer et al., 2008; Li et al., 2023), fluid flow (Galbraith et al., 1998; Helmke et al., 2003), and magnetic tweezers (Bonakdar et al., 2016; Hu et al., 2003). These indirect studies report conflicting results on the fundamental structural mechanics of these cytoskeletons. Some studies describe the cytoskeleton, especially microtubules, as largely discrete cell-scale elements consistent with the cellular tensegrity model (Hu et al., 2003; Ingber et al., 2000; Maniotis et al., 1997; Wang et al., 2001), whereas others observe more continuous and localized structural responses (Bonakdar et al., 2016; Heidemann et al., 1999; Helmke et al., 2003; Ingber et al., 2000). Such inconsistencies may arise from the technical limitations of the experimental methods. The external forces applied on the cell surface reach the intracellular targets by propagating through the actin cortex, which itself is a complicated force generator (Salbreux et al., 2012). These methods generally rely on strong perturbations inducing the cytoskeletal deformations of several micrometers, which are much larger than the endogenous deformations (Ji et al., 2008). To establish a rigorous force-structure relationship of the cytoskeleton, more direct investigations overcoming these limitations are required.

In this study, we perform a comprehensive analysis of how the microtubule and actin cytoskeletons structurally respond to direct intracellular load. Ferrofluid-based intracellular magnetic tweezers reveal the rheological property of the microtubule complex primarily determined by the filamentous actin. The deformations of microtubule and actin, at both network- and single-polymer scales, under different loads are all consistent with a view that individual microtubule polymers are enclosed by an elastic actin body. These results directly evidence a highly integrated nature of the microtubule and actin cytoskeletons throughout the cytoplasm defining their physical behavior as a simple cell-scale continuum.

## Results and discussion

### Direct intracellular load reveals the rheological properties of microtubule complex that depend on actin

**Figure 1.**
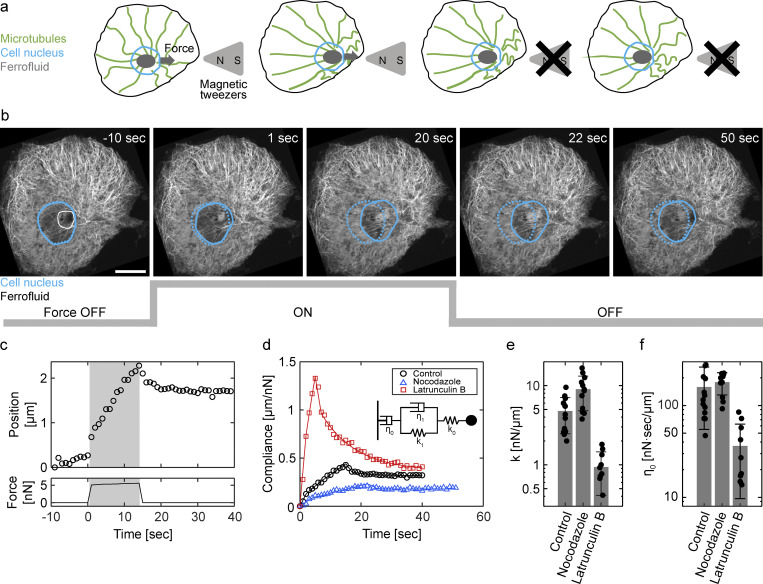
**Actin-dependent rheological properties of microtubule-nucleus complex. (a)** Creep experiment setup. Magnetic tweezers generate high magnetic force on the ferrofluid injected into the nucleus. The applied force continuously displaces microtubule–nucleus complex (creep phase). After releasing the force, the complex partly moves back and eventually stops (relaxation phase). **(b)** Time-lapse confocal images of the microtubule–nucleus complex in the creep experiment. Cyan indicates outlines of the cell nucleus (broken line: nucleus outline at *t* = 0 sec). White line indicates the outline of injected ferrofluid. Scale bar is 10 μm. **(c)** The position of the nucleus (top) and the amplitude of the magnetic force (bottom) are plotted as a function of time. Shaded area indicates the creep phase. **(d and e)** Inhibitor experiments. **(d)** The compliance of control (black), nocodazole (blue), and latrunculin B (red) conditions is plotted as a function of time. Single representative data for each condition is shown. The solid line indicates the fittings using the Burgers model (inset). **(e and f)** The effective spring constant *k* (e) and the long-term viscosity *η*_0_ (f) of control (*n* = 15), nocodazole (*n* = 12), and latrunculin B (*n* = 10) conditions. Error bars indicate the standard deviation.

**Figure S1.**
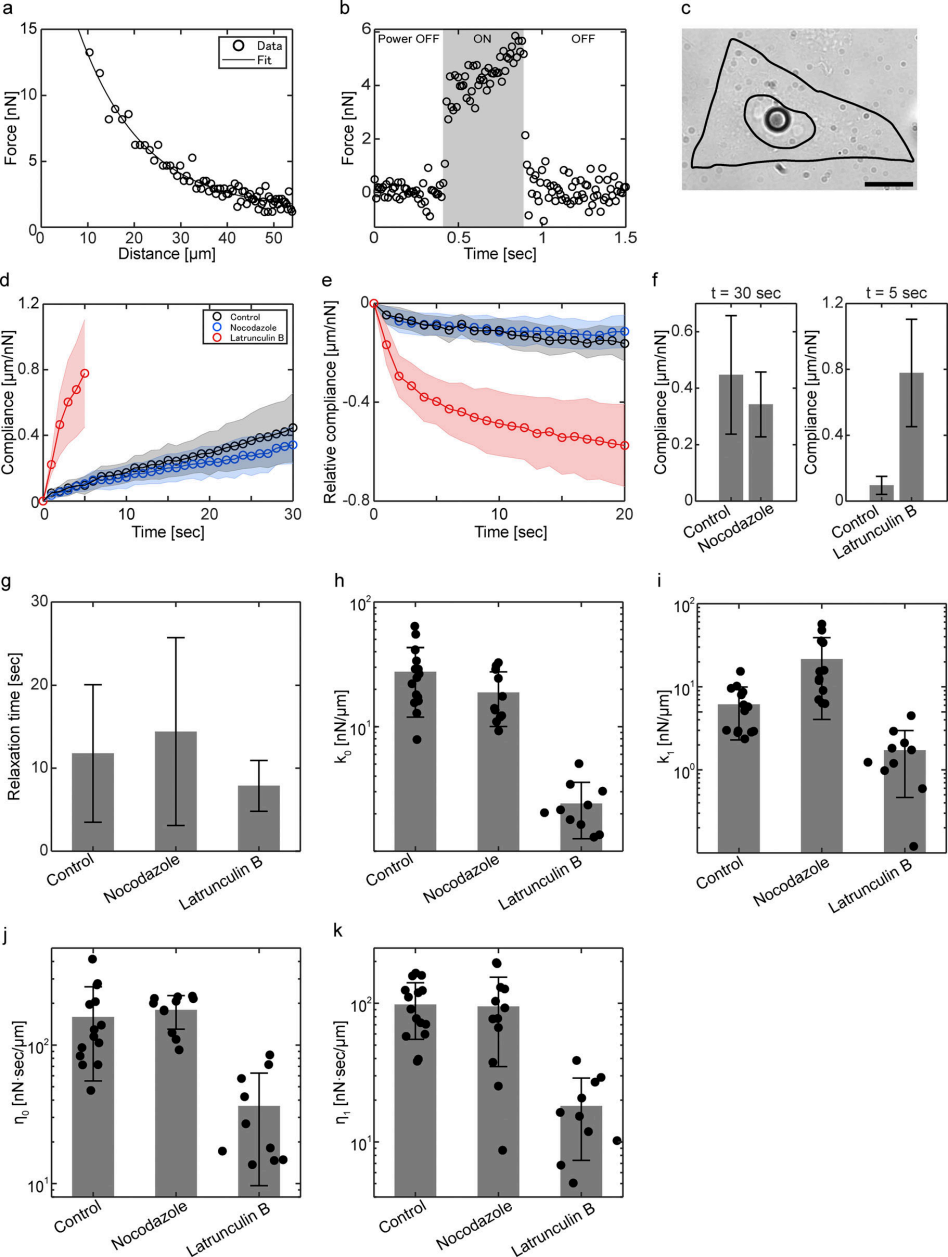
**Characterization of ferrofluid-based magnetic tweezers and extended analysis of creep experiments, related to** Fig. 1**. (a)** Calibrating force–distance relationship of the ferrofluid-based magnetic tweezers. The calibration was performed by tracking a ferrofluid sphere in a high-viscosity test fluid under a given magnetic field (Materials and methods). The solid lines show the fit with an exponential function. **(b)** Characterization of turn-on/off dynamics with high-temporal resolution recording. The time evolution of the magnetic force was recorded at 100 Hz. The time required to turn on/off the magnetic force was around 40 msec. Black shaded area indicates the period where the magnetic field was present. **(c)** A bright field image of a cell which has ferrofluid injected into the nucleus. Scale bar is 10 μm. **(d and e)** Mean compliance in the creep (d) and relaxation (e) phases. Error bar indicates standard deviation. **(f)** Compliance at *t* = 30 and 5 sec in the creep phase. **(g)** Characteristic timescale of the relaxation phase. **(h–k)** Four viscoelastic parameters *k*_0_, *k*_1_, *η*_0_, and *η*_1_ of the microtubule complex determined using the Burgers model.

In an interphase mammalian cell, the majority of microtubules radiate from the centrosome to form a cell-scale astral structure (Bornens, 2008; Burakov et al., 2003). We first aimed to quantify how the interphase microtubule aster responds to direct mechanical load. To this end, we adopted Chinese Hamster Ovary (CHO) culture cells (Brangwynne et al., 2007) and developed a magnetic tweezers methodology. Initial investigation revealed that the forces around 100 pN, which could be archived with standard micron-scale magnetic particles (Anjur-Dietrich et al., 2024; Garzon-Coral et al., 2016; Kollmannsberger and Fabry, 2007; Tanimoto et al., 2018), were not sufficient to induce detectable structural changes in the microtubule asters. Therefore, we established new ferrofluid-based magnetic tweezers that take advantage of the mechanical link between the astral microtubules and the cell nucleus via the centrosome (Burakov et al., 2003; Lombardi et al., 2011). We injected a small amount of PEG-passivated ferrofluid into the nucleus and applied an external magnetic field. In this way, we were able to generate calibrated forces as large as 10 nN inside the nucleus (Fig. 1 a and Fig. S1, a–c; Materials and methods). The large magnetic forces were sufficient to induce consistent movements and deformations of the astral microtubules (Fig. 1 b and Video 1). The nucleus and the microtubule aster moved cohesively throughout the experiments, suggesting that the microtubule–nucleus connection was not grossly influenced by the applied force. The applied intranuclear force induces little deformation of the nucleus of several percent in axial length, which is consistent with the reported nucleus stiffness (Lammerding, 2011). It was also confirmed that the injected cells survived for at least several hours without visible damage. We concluded that this unique setup allowed us to directly characterize the structural mechanics of microtubules in living cells.

**Video 1.** **Time**-**lapse of the microtubules in the creep experiment at 1-s frame intervals.** Scale bar is 10 μm. The movie was rotated to make the direction of the force horizontal.

**Figure S2.**
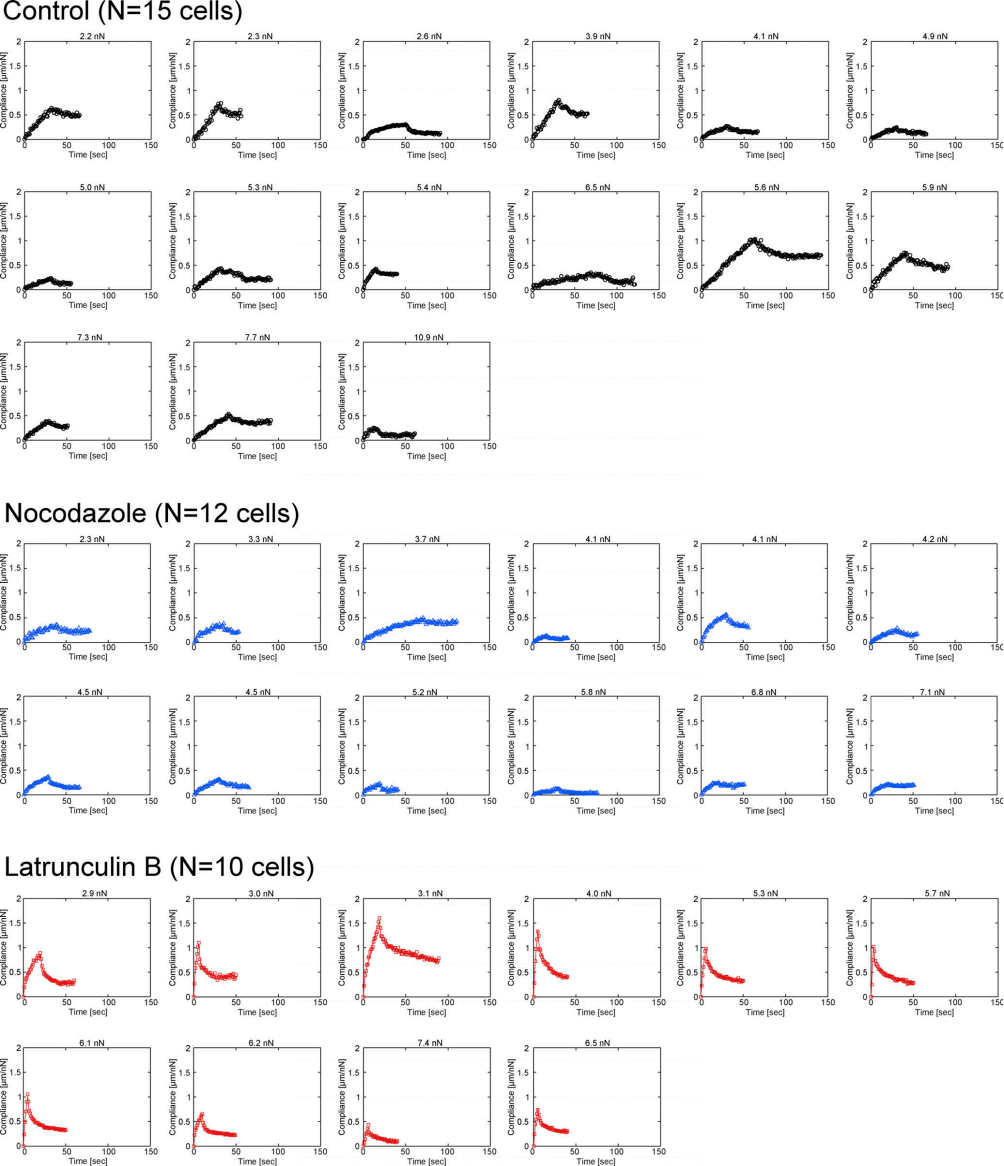
**All individual data of creep experiments, related to** Fig. 1**.** All individual data of creep experiments used in the rheological analysis in Fig. 1, c–e; and Fig. S1, d–k. The time evolution of compliance of all individual data of control (*n* = 15, black), nocodazole treatment (*n* = 12, blue), and latrunculin B treatment (*n* = 10, red) are shown. The graph title indicates the amplitude of applied creep forces.

The direct force application revealed rheological properties of the microtubule-nucleus complex. In the creep experiment presented in Fig. 1 c, a constant force of 5 nN was applied to the microtubule-nucleus complex for about 10 sec (creep phase) and then removed (relaxation phase). At the beginning of the creep phase, the complex was displaced very rapidly from its stationary position around the cell center (Fig. 1 c). In the rest of the creep phase, the complex continued moving with a speed gradually decreasing to a final constant value. In the relaxation phase, the complex suddenly recoiled at the beginning, then it gradually slowed down and eventually stopped. These results suggest that the complex behaves mostly elastic at short timescale and becomes more viscous at longer timescale. These timescale-dependent rheological properties were quantitatively described by the standard Burgers model, in which Maxwell and Kelvin-Voigt elements are connected in series (Materials and methods) (Bausch et al., 1999). Fitting with the model, the effective spring constant *k* = *k*_0_*k*_1_/(*k*_0_+*k*_1_) and the long-term viscosity *η*_0_ of the microtubule-nucleus complex were determined as *k* = 4.76 ± 2.30 nN/μm and *η*_0_ = 158.8 ± 45.5 nN sec/μm (Fig. 1, d–f; Fig. S1, d–k; and Fig. S2).

We then performed inhibitor experiments to investigate cytoskeletal components responsible for the rheological behavior of the microtubule–nucleus complex. The initial hypothesis was that the rheological behavior was mainly defined by the astral microtubules (Garzon-Coral et al., 2016; Tanimoto et al., 2018). We used nocodazole to depolymerize microtubules and repeated the creep experiments. To our surprise, the nocodazole treatment did not significantly change the resistance associated with the intracellular movement of the nucleus (Fig. 1, d–f). This result indicates that most of the magnetic force applied to the nucleus-microtubule complex in normal conditions is counterbalanced by some structures other than the microtubules. We then investigated the roles of actin structure by treating the cells with latrunculin B, which inhibits actin polymerization and disrupts the filamentous actin. This treatment largely decreased the resistance of the microtubule-nucleus complex, and both the effective spring constant and the long-term viscosity decreased from one-third to one-fourth (Fig. 1, d–f). These results suggest that the microtubule–nucleus complex is mechanically supported by cytoplasmic structures, with a major contribution from the filamentous actin.

### Load-induced deformation field of microtubule and actin

**Figure 2.**
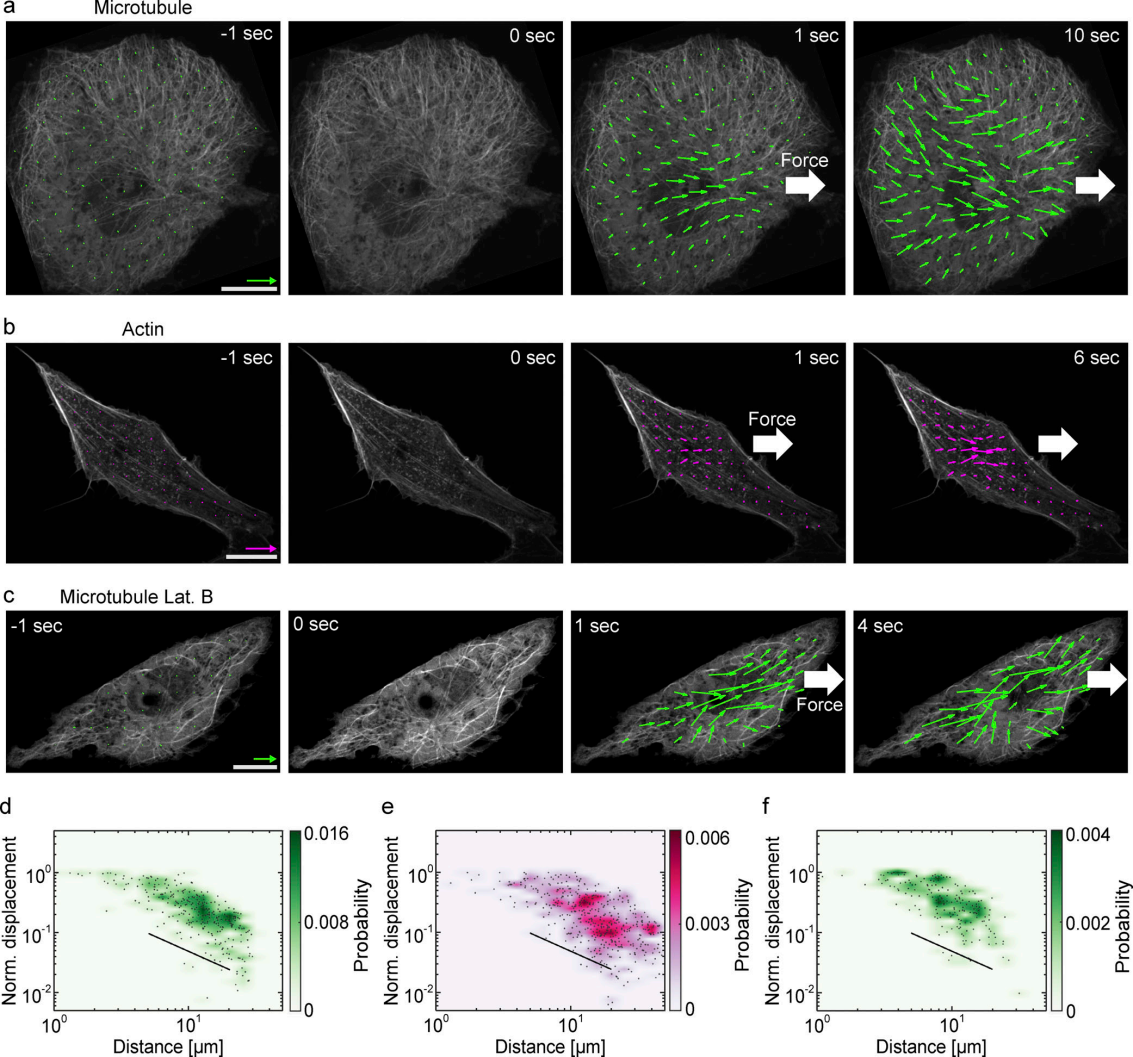
**Load-induced deformation field of microtubule and actin. (a–c)** Deformation analysis of the microtubule and actin cytoskeletons during creep. **(a and c)** The local displacement field of the microtubules (green arrows) is overlayed on a series of confocal images of EGFP-tubulin. **(b)** The local displacement field of the filamentous actin (magenta arrows) is overlayed on a series of confocal images of StayGold-Lifeact. *t* = 0 sec corresponds to the onset of the creep phase. The cells were either in normal conditions (a and b) or treated with latrunculin B (c). Scales are 0.2 μm (color arrow, for the displacement vector) and 10 μm (white bar, for confocal image). **(d–f)** The amplitudes of the local displacements of the microtubules in normal conditions (d), the filamentous actin in normal conditions (e), and the microtubules in latrunculin B condition (f) are plotted as a function of the distance from the load position. The solid line showing a slope of −1 is a guide for the eyes. 285 data points from 6 cells (d), 492 data points from 5 cells (e), 177 data points from 5 cells (f) were analyzed.

**Figure S3.**
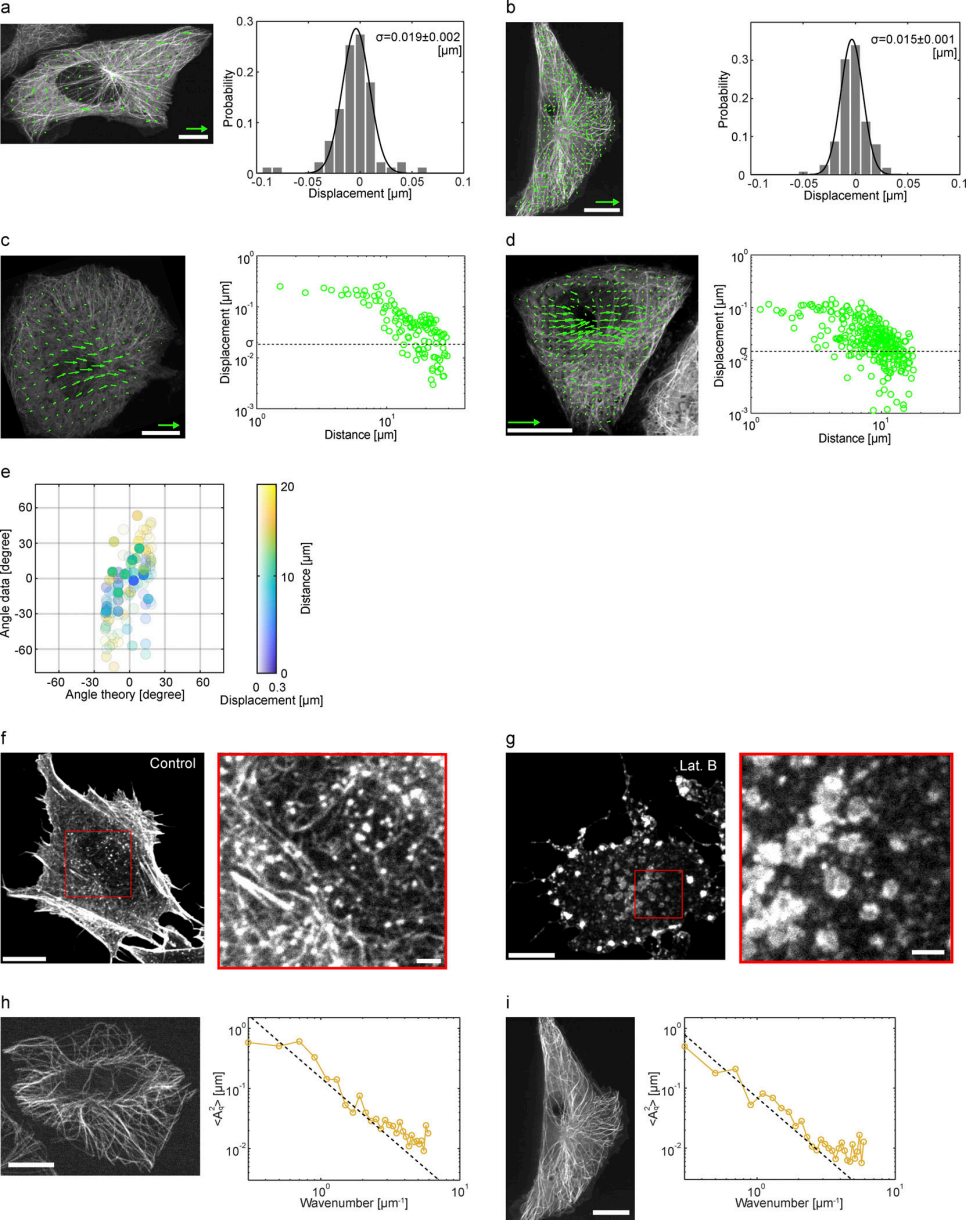
**Characterization of the PIV analysis, the effects of latrunculin B treatment on the organization of filamentous actin, and extended Fourier analysis of unperturbed microtubules, related to** Figs. 2 and 3**. (a–d)** The resolution of the PIV analysis was evaluated by performing the same analysis for control cells which were not subjected to external forces. The analysis was performed for standard confocal (a and c) and super-resolution confocal (b and d). **(a and b)** Left: Two successive snapshots of live CHO cells expressing EGFP-tubulin were acquired at 1 sec interval and analyzed by PIV. The result shows that the detected displacements were significantly small compared to the cells that applied creep forces for the same 1 sec (shown in c and d). Right: Probability of the displacements detected in control. The probability distribution was well fitted with a single Gaussian function with the variance σ= 0.019 ± 0.002 μm (standard confocal) and σ= 0.015 ± 0.001 μm (super-resolution confocal). **(c and d)** Representative single-cell data of the deformation analysis. The local displacement of microtubules of a single cell in a creep experiment is shown with the value of σ. The plot indicates that most of the detected displacements in the creep experiment are above the noise level. Scales are 10 μm for the microscopy image (white bar) and 0.2 μm for the displacement vector (green arrow). **(e)** Orientational analysis of the deformation of microtubules. The angle of microtubule displacement is plotted as a function of the predicted angle from the model. The distance from the load position and the amplitude of the displacement are color coded. **(f and g)** The filamentous actin of control (f) and latrunculin B-treated (g) cells were visualized by Lifeact-StayGold. The actin meshwork observed in control cells was not observed in latrunculin B-treated cells. The imaging condition and image contrast are the same for the two conditions. Scale bars are 10 μm (left) and 2 μm (right enlarged and enhanced). **(h and i)** Fourier analysis of unperturbed microtubule shape using standard (h) or super-resolution (i) confocal microscope. Left: Representative snapshots of microtubules visualized using Tubulin-EGFP. Scale bar is 10 μm. Right: The ensemble-averaged square amplitude of the Fourier modes of microtubule shape plotted as a function of the wavenumber. Broken line indicates slope −2.

We then analyzed the structural changes of microtubule asters under the load. The deformation of microtubule asters in the creep experiments was quantified by determining the local displacement of microtubules with Particle Image Velocimetry (Fig. 2 a; Fig. S3, a–d; and Video 2; Materials and methods). Throughout the creep phase, the local displacement of microtubules had its peak around the nucleus and persistently decayed towards the cell periphery. This decaying profile suggests that the microtubules strongly interact with their surrounding materials in the cytoplasm; otherwise, the applied force should lead to translational movement of the entire microtubule aster and thus the displacement field should be mostly homogeneous.

**Video 2.** **Left: Time**-**lapse of the microtubules in the creep experiment at 1-s frame intervals.** Right: The displacement field of microtubules (green arrow) is overlayed on the same confocal images. Scales are 0.2 μm (arrow, for the displacement vector) and 10 μm (bar, for confocal images).

We also investigated how intracellular actin structures responded to the loads. Lifeact-StayGold labeling filamentous actin highlights a dense meshwork around the microtubule-nucleus complex as well as the stress fibers underneath. We found that the stress fibers did not show visible deformations under the creep, whereas the actin meshwork clearly showed deformations that had their peak around the nucleus and decayed towards the cell periphery in the same way as the microtubules (Fig. 2 b and Video 3). To quantitatively compare the deformation profiles of microtubule and actin, we plotted the local displacement of the two cytoskeletons as a function of the distance from the load position (Fig. 2, d and e). An initial 1 sec of the creep phase was used for the analysis to focus on the short timescale, where the system behaves mostly elastically. The deformation profile was parameterized with a crude fit using a power-law function, *u*(*r*)∼*r*^*α*^, where *u*(*r*) is the amplitude of displacement at the distance *r* from the load position and *α* is a scaling component. The fit gives close values of the scaling components of the two profiles; *α* = −1.09 ± 0.12 for the microtubules and −0.87 ± 0.11 for the actin meshwork (± indicates the standard error in fitting parameters), suggesting that the deformation profiles of the two cytoskeletons share the same characteristics. Taken together, these results suggest that though the microtubules appear to be largely discrete and structured in the cytoplasm, they respond to mechanical load like a continuous body similar to the actin meshwork.

**Video 3.** **Left: Time**-**lapse of the filamentous actin in the creep experiment at 1-s frame intervals.** Right: The displacement field of filamentous actin (magenta arrow) is overlayed on the same confocal images. Scales are 0.2 μm (arrow, for the displacement vector) and 10 μm (bar, for confocal images).

We hypothesized that the microtubules pervade into the dense actin meshwork and that the actin meshwork defines the observed elastic continuum-like behavior of the microtubules (Brangwynne et al., 2006; Robison et al., 2016). Accordingly, we found that disruption of the actin meshwork by latrunculin B led to stretching the deformation profile of microtubules (Fig. 2, c and f; and Video 4). The displacement-distance scaling component was increased in this condition (*α* = −0.83 ± 0.15), showing that the deformation field becomes flatter in the absence of the actin meshwork. This result is consistent with the view that the microtubule and actin cytoskeletons are physically coupled either directly or indirectly, and the coupling largely determines the load response of the microtubules.

**Video 4.** **Left: Time**-**lapse of the microtubule complex in the creep experiment with the presence of latrunculin B at 1-s frame intervals.** Right: The displacement field of microtubules (green arrow) is overlayed on the same confocal images. Scales are 0.2 μm (arrow, for the displacement vector) and 10 μm (bar, for confocal images).

### Shape dynamics of single microtubules under load

**Figure 3.**
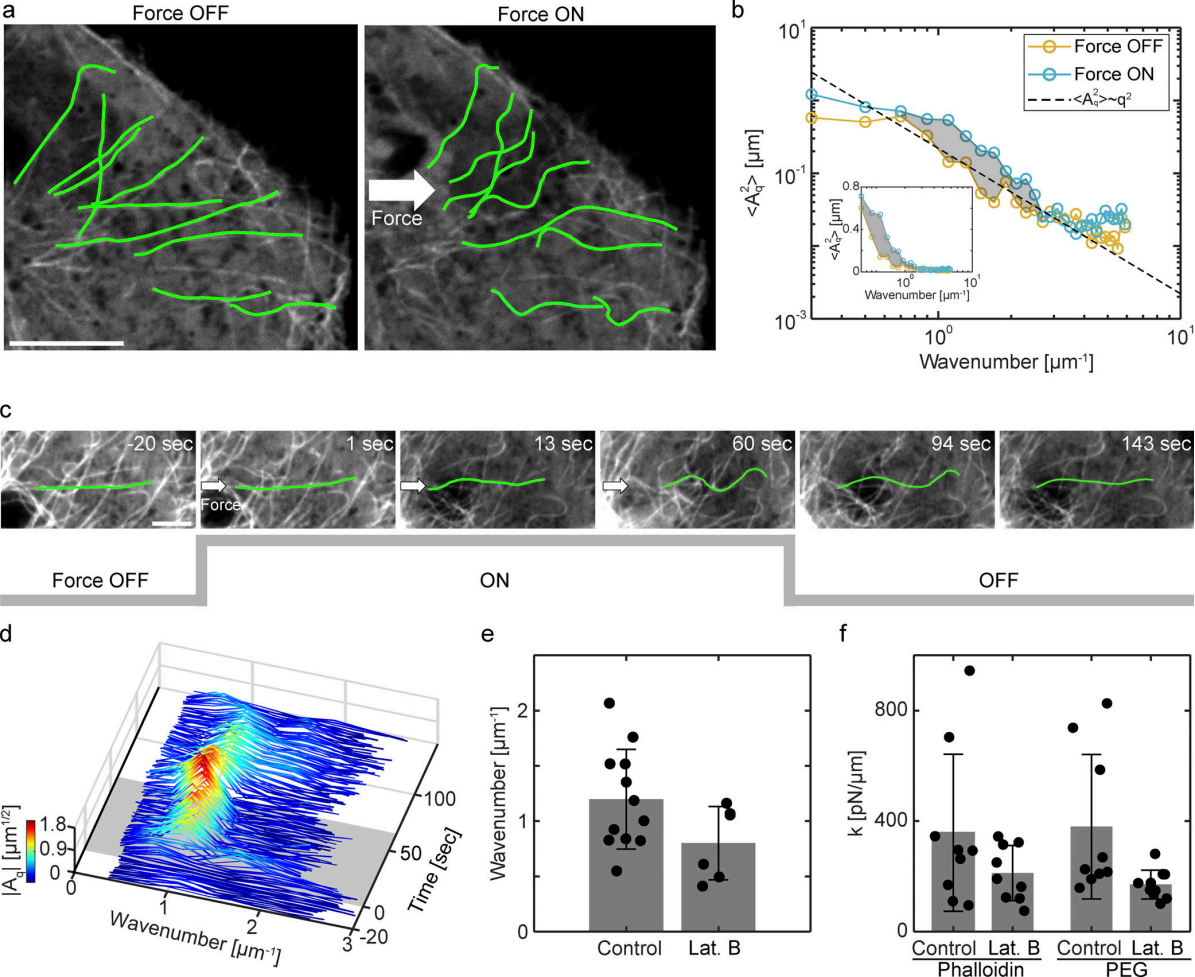
**Shape dynamics of single microtubules under load. (a)** Microtubules before (left) and during (right) compression. Traced microtubule shape (green) is overlayed on confocal images of EGFP-tubulin. Scale bar is 10 μm. **(b)** The ensemble-averaged square amplitude of the Fourier modes of microtubule shape is plotted as a function of the wavenumber. Yellow (blue) color indicates before (under) a compression. Shaded area indicates the difference in the mode amplitude between 0.7 and 3 μm^−1^. More than 100 microtubules from >10 cells were analyzed. Inset: enlarged plot in semi-log scale. **(c–e)** Non-Euler buckling of microtubules. **(c)** Time-lapse images of an axially compressed microtubule. A traced microtubule shape (green) is overlayed on confocal images of EGFP-tubulin. *t* = 0 sec corresponds to the onset of the compression. Scale bar is 2 μm. **(d)** The time evolution of mode amplitudes of the microtubule is shown in c. Shaded area indicates the period of compression. The mode amplitude is also color coded. **(e)** The most unstable wavenumber of axially compressed microtubules in control (*n* = 12 microtubules) and latrunculin B (*n* = 6 microtubules) conditions. **(f)** Effective spring constant of the bulk cytoplasm measured with particle-based magnetic tweezers. Beads coated either with phalloidin (*n* = 11 for control, *n* = 10 for latrunculin B) or PEG (*n* = 9 for control, *n* = 11 for latrunculin B) were used. Error bars indicate the standard deviation.

To further characterize how the microtubule and actin cytoskeletons may interact, we analyzed the shape dynamics of individual microtubules under load. We tracked the dynamical shape of around 100 microtubules in the compressed region during the creep phase. The compressive forces visibly deformed individual microtubules (Fig. 3 a). The change in microtubule shape was quantified by defining the tangential angle *θ* as a function of the contour length *s* along the polymer. The tangential angle *θ*(*s*) was further decomposed into the Fourier modes (Brangwynne et al., 2007; Gittes et al., 1993) as

$$\theta \left(s\right)=\sqrt{\frac{2}{L}}\overset{\infty }{\underset{n=0}{\sum }}A_{q}\cos\left(qs\right),$$

where *L* is the total length of the microtubule and *A*_*q*_ is the amplitude of the Fourier mode with a wavenumber *q* = *n*π/*L*. As reported by Brangwynne et al. (2007), in the normal condition without external load, the dependency of the mode amplitude on the wavenumber was close to $A_{q}^{2}∼q^{-2}$, similar to the shape fluctuation of a rigid polymer at thermal equilibrium (Fig. 3 b; and Fig. S3, h and i). The Fourier analysis shows that the mode amplitude increased under the creep, reflecting that the compressive forces further bent the microtubules. Importantly, the compressive forces mostly increased the mode amplitude around the wavenumber of 1 μm^−1^, rather than shifted up the full spectrum. The result suggests that there is a specific wavenumber at which the microtubules tend to bend in response to compressions.

To investigate how the characteristic wavenumber emerges, we focused on axially compressed microtubules whose shape was nearly straight before the force application. These microtubules were found to buckle in response to the compressive load (Fig. 3, c and d; and Video 5). Strikingly, they underwent higher-order buckling instability. This instability is different from classical Euler buckling, an arc-shaped end-to-end deformation, and suggests that the microtubules are enclosed by elastic materials (Brangwynne et al., 2006; Brodland and Gordon, 1990; Robison et al., 2016). The most unstable wavenumber was determined as ${\tilde{q}}_{\text{control}}=1.19\pm 0.45$ μm^−1^ (*n* = 12 microtubules), in a good agreement with the ensemble analysis (Fig. 3 e). We repeated this experiment with disrupting actin structures using latrunculin B. It was observed that the microtubules still exhibited non-Euler buckling instability, but with longer length-scale. The most unstable wavenumber in actin disruption was ${\tilde{q}}_{\text{actin}-}=0.80\pm 0.33$ μm^−1^ (*n* = 6 microtubules), which was about 20% smaller than that in normal conditions.

**Video 5.** **Time**-**lapse of an axially compressed microtubule at 1-s frame intervals.** Scale bar is 2 μm.

To quantitatively understand these results, we directly evaluated the rheological properties of the environment surrounding the microtubules using standard particle-based magnetic tweezers (Materials and methods). The magnetic probe beads were coated with phalloidin to facilitate their interaction with the actin filaments (Hayakawa et al., 2008). Rheological measurement using the phalloidin beads revealed that latrunculin B treatment decreased the effective elasticity of the cytoplasm by about 50% (Fig. 3 f). The result shows that filamentous actin contributes a major part of the cytoplasmic elasticity. We then performed the same measurement using magnetic beads coated with PEG for passivation (Valentine et al., 2004). We found that the effective elasticity of the cytoplasm in both normal and latrunculin B conditions was mostly the same as the one defined using phalloidin beads (Fig. 3 f). This result indicates that filamentous actin contributes to the cytoplasmic elasticity without specific interactions. The dependence of the most unstable wavenumber $\tilde{q}$ of the microtubules on the bulk elasticity *E* can be expected as $\tilde{q}={\left(E/\kappa \right)}^{1/4}$, where *κ* is the bending modulus of the microtubules (Brangwynne et al., 2006; Landau et al., 1986). Therefore, one can expect ${\tilde{q}}_{\text{control}}/{\tilde{q}}_{\text{actin}-}={\left(E_{\text{control}}/E_{\text{actin}-}\right)}^{1/4}$. We found ${\tilde{q}}_{\text{control}}/{\tilde{q}}_{\text{actin}-}$ was 1.49, which was in good agreement with the measured elasticity of the cytoplasm ${\left(E_{\text{control}}/E_{\text{actin}-}\right)}^{1/4}=1.22$. These results suggest that the microtubules are integrated with the actin meshwork at the single polymer scale.

### Demonstration of the physical integration of microtubule and actin cytoskeletons

**Figure 4.**
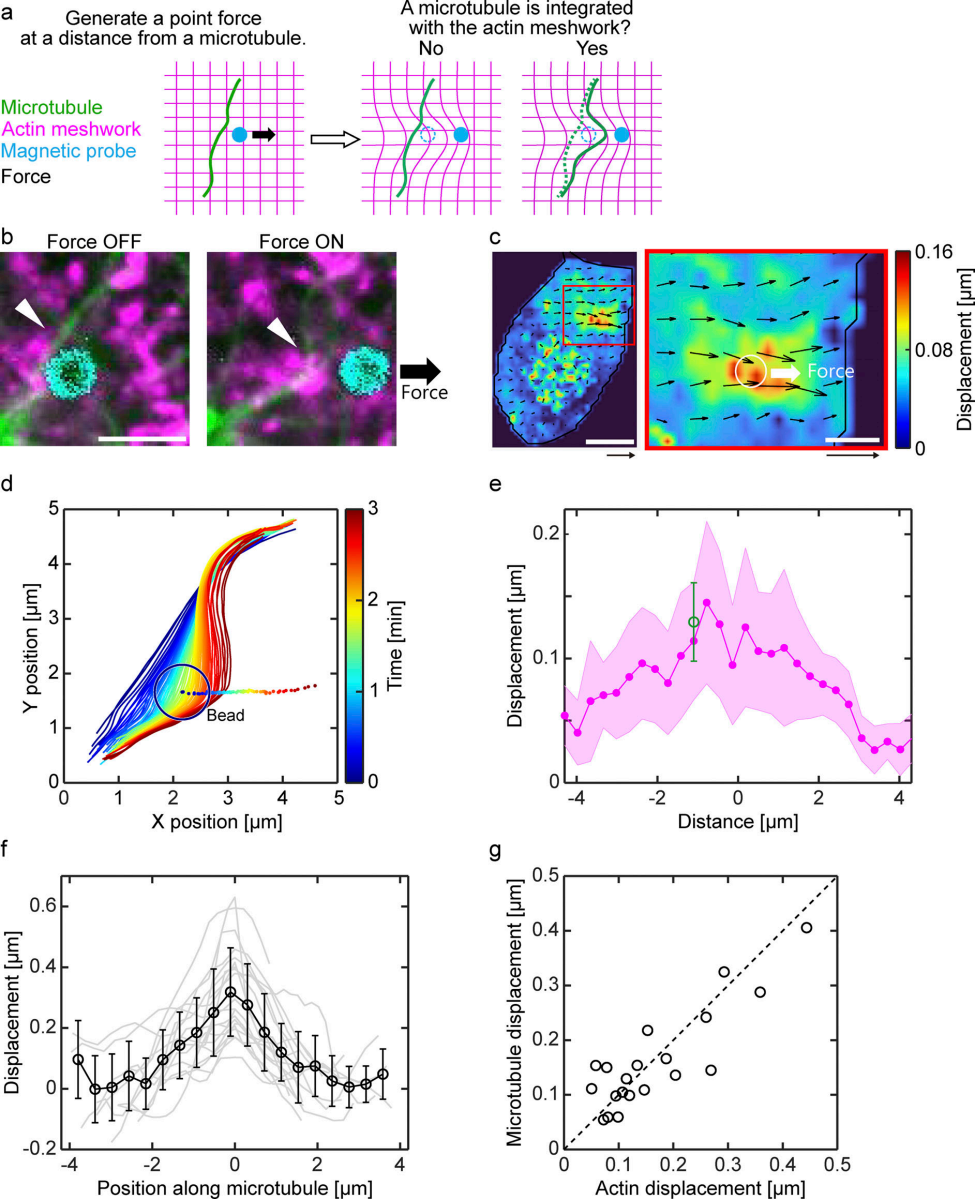
**Demonstration of the physical integration of microtubule and actin cytoskeletons. (a)** Experimental design. A localized force was generated in the actin meshwork a short distance from a microtubule. The force was expected to induce a deformation of the nearby microtubule by propagating through the actin meshwork. **(b)** Snapshots of microtubule (green), actin (magenta), and a phalloidin-coated magnetic bead (cyan) before and during the force application. Arrowhead indicates the analyzed microtubule. Scale bar is 2 μm. **(c)** Local displacement field of actin meshwork. The black arrow and color indicate the vector and amplitude of the displacements, respectively. Scales are 0.2 μm (arrow, for the displacement vector) and 10 μm (bar, for confocal image). Right: An enlarged plot highlighting the position of the bead and the direction of the force (white circle and arrow). Scales are 0.2 μm (arrow, for the displacement vector) and 2 μm (bar, for confocal image). **(d)** Time evolution of the microtubule shape (solid lines) and the bead position (dots). The black circle indicates the outline of the bead at the initial frame. Time is color coded. **(e)** The displacement of actin meshwork (magenta) along the direction of the force is plotted as a function of the distance from the load position. Green circle indicates the displacement of the microtubule. Shaded area and error bar indicate the standard deviation. **(f and g)** Statistics. **(f)** Remote force-induced deformation of single microtubules. The deformation was defined as the local displacement of microtubules during the force application. Solid black line indicates the mean and the standard deviation. **(g)** The maximum displacement of microtubules is plotted as a function of the displacement of actin meshwork at the same position. *n* = 20 measurements from 19 cells.

Lastly, we sought to directly demonstrate the physical integration of the microtubule and actin cytoskeletons. We designed an experiment to generate a localized force in the actin meshwork a short distance from a microtubule (Fig. 4 a). It was expected that the force would propagate through the actin meshwork and deform the nearby microtubule. In the experiment presented in Fig. 4, b–e, a phalloidin-coated small magnetic bead was placed in the actin meshwork ∼1 μm away from a microtubule. The radii of the bead and the microtubule were 0.5 and 0.01 μm respectively, therefore the initial distance between the surfaces of the bead and the microtubule was ∼0.5 μm. A magnetic field was then applied so that the bead moved away from the microtubule. The applied force induced deformations of the actin meshwork in the vicinity (Fig. 4 c). The induced displacement of the actin meshwork took its maximum value (∼0.15 μm per 3 sec of force application) around the bead position and gradually decreased with a distance; so that about 0.05 μm displacement was induced at 3 μm from the bead. As expected, we observed consistent deformation of the microtubule to the direction of the applied force (Fig. 4 d). Strikingly, the displacement of the microtubule was nearly equal to the displacement of the actin meshwork at the same distance from the load (Fig. 4 e). This observation was statistically confirmed by repeated experiments (Fig. 4, f and g). These results quantitatively demonstrate that the microtubule and actin cytoskeletons act as an integrated body in the cytoplasm.

### A novel approach to probe intracellular mechanics at high force regime

The presented study has illustrated the force-structure relationship of the microtubule and actin cytoskeletons using direct intracellular load, to our knowledge, for the first time. The cytoskeleton is remarkably strong and can generate and withstand large forces of tens of nN (Dembo and Wang, 1999; Vignaud et al., 2021). Therefore, to probe their structural mechanics, large mechanical perturbations comparable to the endogenous forces are required. The required 10 nN order force is 10–100 times larger than the limits of conventional intracellular mechanical manipulations including optical tweezers (Guet et al., 2014) and particle-based magnetic tweezers (Anjur-Dietrich et al., 2024; Garzon-Coral et al., 2016; Tanimoto et al., 2018). The presented study thus has introduced a new ferrofluid-based approach, which enables to quantitatively generate 10 nN order forces at the defined location inside living cells.

The introduced method utilizing the cell nucleus as a controllable force-generating probe has advantages and drawbacks. A unique advantage is that the method closely follows a physiological force transmission mode. It is fairly well established that the nucleus and microtubule aster apply mechanical forces to each other through the centrosome (Hale et al., 2011; Lombardi et al., 2011; Maniotis et al., 1997). This direct interaction plays essential roles in many biological processes such as nucleus positioning and cell polarization. Our method transmitting intranuclear magnetic forces to the astral microtubules may recapitulate these physiological mechano-interactions and thus serve as a novel direct tool to study mechanobiology involving the microtubule-nucleus system (Li et al., 2023).

A drawback of the method is on the specificity of the force target. As the same as all other endogenous structures, the nucleus can interact with various intracellular materials through specific and nonspecific modes (Khatau et al., 2009). To avoid possible ambiguity arising from such generic interactions, we restricted the use of the ferrofluid-based technique to characterize robust properties such as the power-law scaling in the cytoskeletal deformation and the thresholding phenomena of microtubule buckling, which are insensitive to the details of the probe forces. For these reasons, we argue that the conclusions drawn by the ferrofluid-based magnetic tweezers should be concrete.

### Structural mechanics of the cytoskeleton

The presented study has revealed a highly continuous behavior of the microtubule cytoskeleton in the cytoplasm. Controlled small perturbations of submicron deformations persistently propagate through the entire microtubule aster until the cell boundary. The strain field of the microtubule aster is largely isotropic and does not depend on the orientation of individual astral microtubules. These results demonstrate that the microtubule aster behaves as a continuous and isotropic material throughout the cytoplasm. This finding sharply contrasts with the standard mechanical picture of microtubule structures that views them as an ensemble of largely discrete stiff polymers (Nédélec, 2002; Tanimoto et al., 2016, 2018).

The rheological analysis shows that the microtubules behave elastically in response to external forces at the timescale of seconds. This is consistent with other micro-rheological measurements showing that the bulk cytoplasm of mammalian culture cells is predominantly elastic (Guo et al., 2014) in contrast to that of some early embryos (Doubrovinski et al., 2017). We propose that this picture may explain the observed constitutive relation of the microtubules. Our analysis of the force-induced deformation field of microtubules suggests that the amplitude of deformation is roughly inversely proportional to the distance (the scaling component close to minus one). Such an inverse relationship between the displacement and the distance is a general feature of an isotropic linear elastic continuum (Landau et al., 1986). We also compared the orientation of the local displacement of microtubules with a simple Green’s function of an isotropic linear elastic body (Fig. S3 e; Materials and methods). The plot shows that the orientation of the measured displacements agrees well with the theoretical prediction. Based on these results, we argue that the simple view of the microtubules as a cell-scale isotropic elastic continuum may quantitatively explain their force-structure relationship. We note that our results do not absolutely exclude more complex material properties such as poroelasticity (Moeendarbary et al., 2013) or plasticity (Bonakdar et al., 2016).

Our results further suggest that the continuum body-like behavior of the microtubules arises from the physical integration with their surrounding cytoplasmic materials including the actin filaments. The physical coupling between the microtubule and actin cytoskeletons has been proposed for decades (Brangwynne et al., 2006; Burakov et al., 2003; Dogterom and Koenderink, 2019; Hale et al., 2011; Robison et al., 2016; Rodriguez et al., 2003; Wang et al., 2001) but has never been tested directly. Thus, a quantitative picture of the interactions and the resulting physical behaviors of the two cytoskeletal systems has not been established. The effects of actin disruption on the force response of the microtubules at both network and single-polymer scales and the force response of the actin meshwork all suggest the highly integrated nature of the two cytoskeletons. Furthermore, we provide direct evidence of the integration by demonstrating that a localized force generated within the actin meshwork propagates to the microtubules at a distance. Our results also suggest that the interaction between microtubule and actin is not entirely rigid. In the ferrofluid-based manipulation where the applied force is directly transmitted to the microtubules, the microtubules deform more than the actin meshwork (Fig. 2, a and b). Conversely, in the particle-based manipulation where the applied force is first transmitted to the actin meshwork, the deformation of microtubules is systematically smaller than that of the actin meshwork (Fig. 4 g). We speculate that these results indicate a frictional interaction between the two cytoskeletons, allowing them to slip to each other. It would be interesting in future studies to precisely characterize the interaction by directly manipulating single microtubule and/or actin polymers in the cytoplasm to obtain a detailed understanding of the nature of the interaction between the two cytoskeletons.

While we have focused on the actin cytoskeleton as a primary determinant of the mechanical behavior of microtubules, our results also highlight the importance of other elements in the cytoplasm. Our analyses of the bulk rheology and the force-induced microtubule buckling suggest that actin and other cytoplasmic materials contribute roughly equally to the elasticity of the environment surrounding the microtubules (Brangwynne et al., 2006). An increasing number of studies describe physical couplings between various intracellular structures including actin-golgi (Guet et al., 2014), microtubule-ER (Kimura et al., 2017), ER-mitochondria (Friedman et al., 2011), and between actin subnetworks (Vignaud et al., 2021). We envision that physical couplings between structures are ubiquitous in the cytoplasm, which evokes an older view of the intracellular space as the protoplasm (Heilbrunn, 1956). The presented study is expected to be instrumental in further studies physically characterizing such inseparable structures, that would lead to a more unified understanding of intracellular organization.

## Materials and methods

### Cells

Chinese Hamster Ovary (CHO) cells were used in all experiments. Wild-type CHO cells were obtained as a kind gift from Dr. Masayuki Murata. CHO cell lines stably expressing EGFP-Tubulin and Lifeact-StayGold were generated in the lab. EGFP-Tubulin plasmid was a kind gift from Dr. Atsushi Suzuki, Lifeact plasmid was a kind gift from Dr. Michael Davidson (plasmid 54491; Addgene), and StayGold(c4)v2.0 plasmid was a kind gift from Dr. Atsushi Miyawaki (plasmid 186296; Addgene) (Hirano et al., 2022). To generate stable cell lines, plasmids were transfected into CHO cells by using lipofectamine LTX (A12621; Thermo Fisher Scientific) following the protocol provided by the company. The cells were cultured in Ham’s F-12 medium (8708335; Wako) supplied with 5% FBS (10270106; GIBCO) and 0.5% penicillin (161-23181; Wako) under 5% CO_2_ at 37°C in a humidified incubator. The cells were sparsely seeded on glass bottom dishes (P50G-0-30; MatTek) coated with fibronectin one day before each experiment.

### Chemicals

Cytoskeletal inhibitions were carried out by directly applying drugs to the culture medium in glass bottom dishes. Nocodazole (31430-18-9; Sigma-Aldrich) was applied 15 min prior to the measurements at a final concentration of 1 μg/ml. Latrunculin B (10010631; Funakoshi) was applied 30 min prior to the measurements at a final concentration of 0.2 μM. For the simultaneous visualization of the microtubule and actin cytoskeletons, cells stably expressing Lifeact-StayGold were treated with Tubulin Tracker Deep Red (T34076; Invitrogen) for 1 h at a final concentration of 0.7 μg/ml.

### Microscopy

Time-lapse image sequences were acquired on a motorized microscope (Ti2-E; Nikon) equipped with a heated stage (ThermoPlate; TOKAI HIT), a 100x PlanApo oil-immersion objective (MRD01905, N.A. 1.45; Nikon), a spinning disk confocal unit (CSU-W1; Yokogawa) with a laser diode illuminator (89 North; LDI-PRIME), and a sCMOS camera (ORCA-FusionBT; Hamamatsu Photonics). The pixel size was 0.064 μm. For super-resolution imaging, the SoRa unit (Yokogawa) with 2.8 magnification was added to the imaging system, leading to the pixel size of 0.023 μm. The equipment was controlled by Micro-Manager (Edelstein et al., 2010). The time interval of time-lapse imaging was set to be 1 sec in the experiments in Figs. 1, 2, and 3 and 3 sec in Fig. 4.

### Magnetic probes

In the ferrofluid-based magnetic tweezers, commercially available ferrofluid (DS-60; SIGMA HI-CHEMICAL) was used. The ferrofluid was mixed with 0.5% wt/wt polyethylene glycol (PEG) oil (008-Fluorosurfactant; RAN Biotechnologies) to promote biological compatibility. The ferrofluid was injected into the cell nucleus using a manual injector (CellTram 4r Oil; Eppendorf) and a micro-manipulator (Injectman 4; Eppendorf). Injection pipettes were prepared from thin wall borosilicate glass capillaries (TW100-4; WPI). The glass capillaries were pulled using a micropipette puller (P-1000; Sutter Instrument) and further ground with a diamond grinder (EG-45; Narishige) to obtain a sharp aperture. Injection pipettes were backloaded with 10 μl of ferrofluid before each experiment and were not re-used. Around 20 fl of ferrofluid was injected into each cell. The injected cells were rested for 1 h in the incubator to recover before the measurements.

In the particle-based magnetic tweezers, paramagnetic particles of 1 μm diameter (Dynabeads MyOne Streptavidin, 65601; Thermo Fisher Scientific ) were used. The surface of the paramagnetic particles was coated following the protocol provided by the companies either with PEG (Rhodamine B-PEG-Biotin, RB-PEG-Biotin 2K; Biochempeg Scientific) for passivation or with phalloidin (Biotin-XX Phalloidin, 00028; BIOTIUM) to facilitate interactions with filamentous actin. After functionalization, the particles were further treated with a biotinylated fluorescent dye (ATTO488-Biotin [AD488-71] or ATTO550-Biotin [AD550-71], ATTO-TEC) for labeling. The paramagnetic particles were injected into the cytoplasm using a motorized injector (Injectman 4; Eppendorf) and the micro-manipulator. One to three particles were injected for each cell. The injected cells were rested for 1 h in the incubator to recover before the measurements.

### Magnetic tweezers

The magnetic tweezers were built in the laboratory following the literature (Kollmannsberger and Fabry, 2007). A cylindrical permalloy with a diameter of 3 mm (Nilaco) was used as a core of the magnetic tweezers. One end of the core was precisely tapered with a grinder to have a tip radius of 2 μm. The core was inserted in a solenoid with 200 or 400 windings. DC stabilized power supplies (PMX18-5A or PMX35-3A; KIKUSUI) were used to generate currents in the solenoid. The position of the magnetic tweezers was controlled with the motorized micro-manipulator mounted on the microscope.

Calibration of the ferrofluid-based magnetic tweezers was performed by analyzing the motion of ferrofluid under magnetic fields in a test fluid with known viscosity (Tanimoto et al., 2018). A spherical ferrofluid with a diameter of 4.5 μm, which is the size of the ferrofluid spheres used in the experiments (4.58 ± 0.98 μm), was placed in a high viscosity medium of Glycerin (dynamic viscosity of 1.412 Pa sec at 20°C). Magnetic tweezers were placed 100 μm away from the ferrofluid sphere. The applied current was set to be 1 A. The motion of the ferrofluid sphere was imaged at 100 Hz to determine its position and speed. From the measured speed, we calculated the force acting on the ferrofluid sphere at a given distance from the tweezers’ tip using Stokes’ law (Fig. S1 a). The global motion of the test fluid during the force application was monitored using non-magnetic tracer beads and found to be mostly negligible. The force-distance relationship of the magnetic tweezers was approximated with an exponential function to define the magnetic force at a given distance.

### Creep experiments

In the creep experiments, the magnetic tweezers were placed close to the cells which had ferrofluid injected into the nucleus. The distance between the cell boundary and the magnetic tweezers’ tip was set to be <10 μm to generate a large magnetic field gradient inside the cells. With this setup, a constant force of 5–20 nN could be stably applied to the cell nucleus by supplying a current of 1 A to the solenoid of the magnetic tweezers. The time required to switch on/off the magnetic force was around 40 msec (Fig. S1 b). The creep force was kept for 10–60 sec which led to the displacement of the microtubule-nucleus complex around 2 μm in normal conditions. The change of the magnetic force during a single measurement was <10%.

### Bulk rheology measurements

The setup of magnetic tweezers was the same in the creep experiments. With this setup, around 100 pN force could be applied for the 1 μm paramagnetic beads injected in the cytoplasm. The force was applied for 10–100 sec, which led to the displacement of the magnetic particles around 3 μm in normal conditions. The change of the magnetic force during a single measurement was <5%. The results were analyzed using the Burgers model (detailed in Analysis 1) to determine the effective spring constant of the bulk cytoplasm.

### Analysis (1) rheological properties of microtubule-nucleus complex

The position of the microtubule-nucleus complex was defined as the position of the nucleus, which could be tracked either with transmitted light or fluorescent images. The compliance of the microtubule–nucleus complex was defined as the position divided by the creep force. The characteristic timescale of the recovery was defined by fitting the compliance in the relaxation phase using a single exponential function with an offset as a fitting parameter. To determine the viscoelastic properties of the microtubule-nucleus complex, the motion of the complex was analyzed using the Burgers model, in which a Maxwell element and a Kelvin–Voigt element are connected in series (Fig. 1 d, inset). In the Burgers model, the time evolution of the position *x*(*t*) during the creep phase (with a constant force *F*) and the relaxation phase (after removing the creep force) is described as Bausch et al. (1999).

$$\frac{x\left(t\right)}{F}=\frac{1}{k_{0}}+\frac{1}{k_{1}}\left(1-e^{-t/\tau_{1}}\right)+\frac{t}{\eta_{0}}\;$$

(creep phase),

$$\frac{x\left(t\right)}{F}=\frac{1}{k_{1}}\left(1-e^{-t_{\text{off}}/\tau_{1}}\right)e^{-\left(t-t_{\text{off}}\right)/\tau_{1}}+\frac{t_{\text{off}}}{\eta_{0}}$$

(relaxation phase),where $\tau_{1}≔\eta_{1}/k_{1}$ is the characteristic timescale of the Kelvin-Voigt element. *t* = 0 (*t* = *t*_off_) corresponds to the beginning (end) of the creep phase. The viscoelastic coefficients were determined by fitting the time evolution of the position of the microtubule-nucleus complex with the above equations. The effective stiffness *k* was defined by $k^{-1}=k_{0}^{-1}+k_{1}^{-1}$. All individual data including pharmacological inhibitor experiments are presented in Fig. S2.

### Analysis (2) deformation field of microtubule and actin cytoskeletons

The local displacement fields of microtubule and actin induced by the creep forces were defined by applying Particle Image Velocimetry (PIV) for the confocal time-lapse images. The initial 1 sec of the creep phase was used for the deformation analysis to focus on the short timescale, where the microtubule complex behaves mostly elastic. The following analysis was performed using the PIVlab tool (Stamhuis and Thielicke, 2014) in Matlab (RRID:SCR_001622). The image of cells right before the application of the creep force was used as a reference. The image sequences were analyzed by computing the cross-correlation between the data and the reference images using three interrogation square windows with a width of 64-, 32-, and 16 pixels with an overlapping of 50%. The final window size corresponds to 1.02 μm. Using control cells without external forces, the resolution of the PIV analysis was estimated as around 20 nm (Fig. S3, a–d).

The amplitude of the local displacement *u* was analyzed by plotting it as a function of the distance from the load position *r*. The amplitude was normalized with the largest value for each cell for further analysis. The coefficient α in the scaling relationship *u*(*r*)∼*r*^*α*^ was determined with a linear fit of the displacement-distance plot in log-log scale.

The orientation of the displacement vectors was compared with a linear elastic body model. We considered deformations of an elastic body of half space induced by a tangential point force on the surface. We set the origin of the coordinate to be the force position and defined an axis along the force direction. Let *ϕ* be the azimuthal angle of a position on the surface. Then, according to the Boussinesq solution (Hu et al., 2003; Landau et al., 1986), the azimuthal angle of the displacement vector at the position *Φ* is given by

$$\Phi =\arctan\left(\frac{\sin\phi \cos\phi }{1+\cos^{2}\phi }\right)$$

*Φ* was compared with the experimentally determined angle of the displacement vector of microtubules (Fig. S3 e).

### Analysis (3) shape dynamics of single microtubules

The contours of single microtubules were semi-automatically tracked using an ImageJ (RRID:SCR_00307) plug-in J-filament (Smith et al., 2010). The plug-in allowed us to determine the cartesian coordinates of the backbones of microtubules in an interactive manner. Using the backbone, the local tangent of the microtubules *θ* was determined as a function of the contour length *s* from one end. Then, the microtubule shape was decomposed into Fourier modes by expressing *θ*(*s*) as a sum of cosines as (Brangwynne et al., 2007; Gittes et al., 1993)

$$\theta \left(s\right)=\sqrt{\frac{2}{L}}\sum_{n=0}^{\infty }A_{q}\cos\left(qs\right),$$

where *L* is the total length of the microtubules, *A*_*q*_ is the Fourier amplitude, and *n* is the mode number. In the ensemble analysis presented in Fig. 3 b, >100 microtubules in a compressed region were analyzed and their Fourier amplitudes of microtubules were averaged by binning with a bin size of 0.2 μm^−1^.

### Statistics

Mean values are given with standard deviation unless specified. Outliers are determined by the Grubbs test. Sample number *n* indicates the number of individual cells unless specified. All measurements were repeated at least three times as independent experiments.

### Online supplemental material

This manuscript contains three supplemental figures and five supplemental videos. Fig. S1 shows characterization of ferrofluid-based magnetic tweezers and extended analysis of creep experiments, related to Fig. 1. Fig. S2 shows all individual data of creep experiments, related to Fig. 1. Fig. S3 shows characterization of the PIV analysis, the effects of latrunculin B treatment on the organization of filamentous actin, and extended Fourier analysis of unperturbed microtubules, related to Figs. 2 and 3. Video 1 shows time-lapse of the microtubules in the creep experiment at 1 sec frame intervals. Video 2 shows time-lapse of the microtubules in the creep experiment at 1 sec frame intervals. Video 3 shows time-lapse of the filamentous actin in the creep experiment at 1 sec frame intervals. Video 4 shows time-lapse of the microtubule complex in the creep experiment with the presence of latrunculin B at 1 sec frame intervals. Video 5 shows time-lapse of an axially compressed microtubule at 1 sec frame intervals. Scale bar is 2 μm.

## Data Availability

The data and materials used in this study are available from a corresponding author (H. Tanimoto) upon request.
